# Differential tolerance capacity to unfavourable low and high temperatures between two invasive whiteflies

**DOI:** 10.1038/srep24306

**Published:** 2016-04-15

**Authors:** Na Xiao, Li-Long Pan, Chang-Rong Zhang, Hong-Wei Shan, Shu-Sheng Liu

**Affiliations:** 1 Ministry of Agriculture Key Laboratory of Agricultural Entomology, Institute of Insect Sciences, Zhejiang University, Hangzhou 310058, China; 2 Institute of Plant Protection, Guizhou Academy of Agricultural Sciences, Guiyang 550006, China

## Abstract

Thermal response and tolerance to ambient temperature play important roles in determining the geographic distribution and seasonal abundance of insects. We examined the survival and performance, as well as expression of three heat shock protein related genes, of two species of invasive whiteflies, Middle East-Asia Minor 1 (MEAM1) and Mediterranean (MED), of the *Bemisia tabaci* species complex following exposure to a range of low and high temperatures. Our data demonstrated that the MED species was more tolerant to high temperatures than the MEAM1 species, especially in the adult stage, and this difference in thermal responses may be related to the heat shock protein related genes *hsp90* and *hsp70*. These findings may assist in understanding and predicting the distribution and abundance of the two invasive whiteflies in the field.

Temperature is one of the most critical abiotic determinants of geographic distribution and seasonal abundance of ectotherms, including insects, on the earth^1^,^2^. Because their body temperature is largely determined by ambient temperature, virtually all biochemical and physiological processes of insects, and consequently the capacity of survival, reproduction and dispersal, vary with changes in temperature^3^. Theoretical and experimental studies have shown that the capacity to tolerate extreme temperatures is probably the most important abiotic factor determining the geographic distributions of most ectotherms including insects^4^,^5^. When it comes to biological invasion, the capacity to tolerate low and high temperatures may become a critical factor in determining the potential geographic range in which the alien species may become established^6^. Within the range of natural distributions, differential responses to extreme temperatures may play a key role in shaping the patterns of relative seasonal abundance between ecologically closely related species, such as different species of aphids or whiteflies colonizing the same habitats^7^,^8^.

The whitefly *Bemisia tabaci* (Gennadius) is a species complex consisting of many morphologically indistinguishable but reproductively isolated cryptic species^9^,^10^,^11^. Whiteflies of this species complex may impose serious damage to a range of important crops worldwide such as cotton, tobacco and many vegetables and ornamental plants, through direct feeding and transmission of over 200 plant viruses mainly begomoviruses^9^,^12^. Within this species complex, two cryptic species, Middle East-Asia Minor 1 (MEAM1) and Mediterranean (MED), formerly often referred to as the B and Q “biotypes”, have attracted much research and management effort in recent years, due to their rapid invasions around the world in the past 30 years^9^,^13^,^14^. In China, MEAM1 was first detected in Shanghai in 1995, and MED was first detected in Yunnan in 2003^15^,^16^. Immediately after their first detection in China, both species were soon found in many other regions of the country, and in many regions of invasion they have been displacing the indigenous species of the whitefly complex^17^,^18^,^19^,^20^, and in some regions, especially in the northern part of the country, MED was found to replace the earlier invader MEAM1^17^,^21^,^22^,^23^. The competition and displacement between MEAM1 and MED have been investigated in relation to a number of factors, such as insecticide susceptibility, behavioural interactions and host plants^23^,^24^,^25^,^26^,^27^.

The effect of temperature on the performance of the MEAM1 and/or MED whiteflies has been investigated by many authors. The two species of whiteflies were usually able to complete development from egg to adult in the temperature range of 15–35 °C though survival was usually substantially reduced at temperatures <20 °C or >30 °C^28^,^29^,^30^,^31^. The percentages of survival of the whiteflies at unfavourable low and high temperatures were further affected by host plants. For example, Tsueda and Tsuchida^32^ showed that at 35 °C MEAM1 and/or MED whiteflies achieve similar survival of ca. 20% from egg to adult on cucumber, but on tomato MEAM1 achieved a significantly higher survival of 32% compared to 2% of MED^32^. Han *et al.*^33^ demonstrated that the MED whitefly achieved ca. 30% and 40% survival from egg to adult at 15 °C when reared on eggplant and bell pepper but no survival when reared on oriental melon^33^. Some attempts have also been made to examine the capacity of tolerance of the two species of whiteflies to sublethal high temperatures, and the data indicate that the two species could usually obtain some survival following a few hours exposure to temperatures in the range of 40–45 °C^34^,^35^,^36^,^37^. However, currently available case studies provide yet insufficient knowledge of the differences between MEAM1 and MED in their thermal tolerance, because in many cases the experiments were conducted on one of the two species at a time and due to the effects of various factors, such as differences of environmental variables other than temperature, data obtained by different authors may not be compared directly^34^,^36^. When trials on thermal tolerance were conducted on the two species simultaneously under one experimental set-up, little attention was given to the variation of thermal response in different developmental stages of the insects^35^,^38^. Insect thermal tolerance is known to vary considerably between life stages in the same species^4^. In addition, thermal tolerance may vary in different geographic populations of the same species^39^,^40^,^41^.

In the present study, we compared the survival and reproduction of the MEAM1 and MED whiteflies from China following exposure of different life stages of the insects to low and high temperatures. We conducted a range of experiments to compare temperature tolerance between MEAM1 and MED whiteflies from Zhejiang, China, that have been maintained in the laboratory for several years. We also included in some of the experiments populations of the two species, which were collected from Guangdong, China and had been reared in the laboratory for only a few generations. In addition, we analyzed the expression of heat shock protein related genes of the two species of whiteflies from Zhejiang. Our objective was to quantify the differences in tolerance to extreme temperatures between the two important species of invasive whiteflies, especially for the populations of these invasive whiteflies in China.

## Materials and Methods

### Insects and plants

Two whitefly species, MEAM1 (*mtCOI* GenBank accession no. GQ332577) and MED (*mtCOI* GenBank accession no. GQ371165), each with two populations, one from Zhejiang and the other from Guangdong, China were used in the experiments. The MEAM1 population from Zhejiang was collected from eggplants in Rui’an (27°48′20′′N, 120°39′57′′E) in September 2008, and had been maintained in the laboratory for 50–60 generations when used in the experiments. The MED population from Zhejiang was collected from pepper plants in Ningbo (29°56′34′′N, 120°40′45′′E) in June 2009, and had been maintained in the laboratory for 40–50 generations when used in the experiments. The MEAM1 population from Guangdong was collected from bell pepper plants in the South China Botanic Garden (23°55′31′′N, 113°21′10′′E), and the MED populations from Guangdong was collected from eggplants in Maoming (21°55′4′′N, 110°50′5′′E) in September 2013. The two whitefly populations of Guangdong had been reared in the laboratory for 1–3 generations when used in the experiments. All the four whitefly populations were maintained on cotton (*Gossypium hirsutum* cv. Zhemian 1793) in insect proof cages (40cm × 50cm × 50cm) in climate–controlled rooms at 26–28 °C, 14:10 light/dark (light: 6:30–20:30), and 60–80% relative humidity. Each of the four laboratory populations was monitored every three generations to detect and avoid any contamination from other whitefly species in the laboratory, using PCR-restriction fragment-length polymorphism analysis and *mtCOI* sequencing as described previously^42^.

All cotton plants used to maintain whitefly populations and conduct experiments were planted singly in 1.5 liter pot with a potting mix (peat moss, vermiculite, organic fertilizer, perlite in a 5:1:1:1 ratio by volume) in insect proof glasshouses at 24–28 °C, 14:10 light/dark (light: 6:30–20:30), 60–80% relative humidity. The plants were used when they reached the 5–7 fully expanded true leaf stage. All plants were visually checked using a 20× hand-lens prior to be used, to ensure that only insect-free plants were used.

Temperature exposure experiments were conducted in climatic chambers (Sanyo, MLR-350H, Sanyo Electric Co., Ltd., Osaka, Japan) which offered precise control of temperatures within ±0.5 °C of the set value. All temperature regimes were set with 60–80% relative humidity and a photoperiod of 14 h light (6:30–20:30) and 10 h darkness.

### Survival of MEAM1 and MED eggs following exposure to high temperatures

This part of the experiments was conducted with MEAM1 and MED whitefly adults from Zhejiang. On the day prior to temperature exposure, cotton plants with red-eye pupae were placed into new insect proof cages after removal of all the adults on the plants. The next day, for each of the two species, approximately 1000 newly emerged adults were collected from the cotton plants and released into a new cage with fresh cotton plants inside, in which the adults were reared for three days to offer sufficient time for the females and males to mate. Then, the female adults were collected and placed onto leaves of new cotton plants, enclosed in clip cages, 10 females per cage. The females were left on the leaves for 24 for them to oviposit and then discarded. We had five clip cages (replicates) for each of the two species. To minimize the effect of leaf position on the plant, we placed two clip cages on each leaf, one for MEAM1 and one for MED. The whitefly eggs, 52–70 eggs per clip cage (replicate), with the cotton plants were then exposed to 37 °C, 39 °C, 41 °C, 43 °C, 45 °C (high temperatures) and 26 °C (control) respectively for 2 h and then all reared at the control temperature of 26 °C. The number of live eggs, from which nymphs had hatched, and dead eggs in each replicate was counted 14 days later when no more hatching was assumed.

### Survival of MEAM1 and MED pupae following exposure to high temperatures

This part of the experiments was conducted with MEAM1 and MED whiteflies from Zhejiang. Nineteen days prior to the temperature exposure, for each of the two species, approximately 500 adults at the age of three days post-emergence were collected using the method described above and released into a new cage with fresh cotton plants inside to lay eggs for 24 h. The adults were then discarded and the newly laid eggs on the plants were reared at 26 °C for 18 days when most of eggs had developed into the red-eye pupal stage. Cotton leaves with pupae, 30–40 pupae per leaf (replicate), were then detached from the plants and placed individually in Petri dishes. The leaves, with the leaf undersurface and whitefly pupae on it facing up, were placed onto the bottom of the Petri dishes, which was covered with 1% agarose gel to provide moisture to the leaves. The Petri dishes were covered with gauge to facilitate rapid homogenization of the temperature inside with ambient temperatures. The Petri dishes with the leaves and whitefly pupae inside were exposed to 37 °C, 39 °C, 41 °C, 43 °C, and 45 °C (high temperatures) and 26 °C (control), respectively, for 6 h and then were transferred to the control temperature of 26 °C. We had five Petri dishes (replicates) for each of the two species. Five days later, the numbers of live pupae, from which whitefly adults had emerged, and dead pupae in each of the replicates were counted.

### Survival of MEAM1 and MED adults following exposure to low temperatures

This part of the experiments was conducted with MEAM1 and MED whiteflies from Zhejiang. On the day prior to temperature exposure, for each of the two whitefly populations, cotton plants with red-eye pupae were placed into new insect proof cages after removal of all the adults on the plants. The next day newly emerged whitefly adults were collected from the cotton plants and placed into 20 ml glass tubes, 50 adults per tube. The opening of the tubes was covered with gauze to allow ventilation and rapid homogenization of temperature inside the tube with ambient temperatures. The tubes holding the adults were then randomly allocated to three treatments at low temperatures of 0 °C, 4 °C and 8 °C and a control temperature of 26 °C, respectively, with 5 tubes (replicates) at each of the temperatures. Based on the data of a preliminary trial, the duration of exposure to the low temperatures was conducted for 48 h. Immediately following the temperature exposure, the tubes with adults were transferred to 26 °C to allow the adults to recover for 2 h. The live and dead adults in each of the tubes were then counted to assess mortality. Adults that could not move when prodded gently with a brush were regarded as dead.

### Survival of MEAM1 and MED adults following exposure to high temperatures

This part of the experiments was conducted with MEAM1 and MED whiteflies from both Zhejiang and Guangdong. The methods for obtaining adults for the test and temperature exposure treatment were the same as that of above for low temperature exposure. In all, we had four whitefly populations. For each of the whitefly population, the tubes holding the adults were randomly allocated to five treatments at high temperatures of 37 °C, 39 °C, 41 °C, 43 °C and 45 °C and a control temperature of 26 °C, respectively, with 10 tubes (replicates) at each of the temperatures. Based on the data of a preliminary trial, the duration of exposure to the high temperatures was conducted for 2 h. Immediately following the exposure, the tubes with adults were transferred to 26 °C to allow the adults to recover for 2 h. The live and dead adults in each of the tubes were then counted and sexed under microscope to assess mortality and sex ratio.

### Fecundity and performance of offspring following exposure of MEAM1 and MED adults to low temperatures

This part of the experiments was conducted with MEAM1 and MED whiteflies from Zhejiang. Four days prior to the temperature exposure, for each of the two whitefly populations, cotton plants with red-eye pupae were placed into a new insect proof cage after removal of all the adults on the plants. Forty-eight hours later, the newly-emerged adults were collected and transferred to a new cage with fresh cotton plants, where they were maintained for another 48 h to offer them sufficient time to mate. Then 100 mated females were randomly collected from each of the two populations and placed into 10 tubes (replicates). Five of the tubes with the female adults were exposed to 4 °C and the other five to the control temperature of 26 °C, for 2 h. Following the exposure, the 10 females in each of the tubes were transferred to a cotton leaf enclosed by a clip-cage. To minimize the possible of effect of leaf position, two clip cages were placed on each cotton leaf, one with MEAM1 adults and other with MED adults. The female adults were kept on the leaves for 48 h for them to oviposit at 26 °C and then discarded. The newly laid eggs were then reared on the cotton plants until adult emergence of the offspring at 26 °C. Fourteen days later, when the individuals with fastest development had reached the fourth instar, the number of dead eggs and live nymphs were recorded. Number of newly emerged adults from each clip cage were counted and sexed daily until no more emergence was observed. From these records, the number of eggs laid by the test adults, the number of eggs that hatched, the development time from egg to adult, and the sex ratio of the offspring were calculated.

### Fecundity and performance of offspring following exposure of MEAM1 and MED adults to high temperatures

This part of the experiments was conducted with MEAM1 and MED whiteflies from both Zhejiang and Guangdong. The basic methods for obtaining adults for the test and temperature exposure were the same as above. In all, we had four whitefly populations. For each of the whitefly populations, the tubes holding the adults were randomly allocated to five treatments at high temperatures of 37 °C, 39 °C, 41 °C, 43 °C and 45 °C and a control temperature of 26 °C, respectively, with 10 tubes (replicates) at each of the temperatures. Based on the data of a preliminary trial, the duration of exposure to the high temperatures was conducted for 2 h. Immediately following the exposure, the 10 females in each of the tubes were transferred to a cotton leaf enclosed by a clip-cage. To minimize the possible of effect of leaf position, two clip cages were placed on each cotton leaf, one with MEAM1 adults and other with MED adults. The female adults were kept on the leaves for 48 h for them to oviposit and then discarded. The observation for the number of eggs laid and performance of the offspring were the same as above for the exposure to low temperature.

### Effect of high temperature on the expression of heat shock protein related genes

This part of the experiments was conducted with MEAM1 and MED whiteflies from Zhejiang. For each of the two whitefly populations, newly emerged whitefly adults (24 h post-emergence) were put into tubes, 50 adults per tube, and then the tubes with the whiteflies inside were exposed to 41 °C for 2 h as described above. Immediately after the exposure, live adults were collected and stored at −80 °C for subsequent gene expression analysis. Total RNA of whitefly was extracted with TRIzol (Invitrogen, USA), and was reverse transcribed using PrimeScript RT reagent Kit (Takara, Japan) after the concentration and purity of RNA samples were examined by nanodrop (Thermal, USA). The expression levels of three heat shock protein related genes including *hsp40*, *hsp70* and *hsp90* were analyzed using SYBR Premix Ex Taq II (Takara, Japan) and CFX96™ Real-Time PCR Detection System (Bio-Rad, USA). Actin served as an internal control, and all the primer used can be found at Mahadav *et al.*^38^.

### Statistical analysis

All percentage data were arcsine square root transformed for use in statistical analysis and back-transformed for presentation in tables and figures. Comparisons of survival of eggs, pupae and adults between the two whitefly species following exposures to each of the low and high temperatures were performed by an independent-samples Student-*t* Test. The number of eggs laid, percentages of the egg hatch, developmental time of offspring and sex ratio of offspring of the populations of the two whitefly species following exposure of adults to low or high temperatures were analyzed by a two-way analysis of variance (ANOVA) followed by Fisher’s least significant difference (LSD) test. Likewise, the levels of expression of each of the heat shock protein related genes were analyzed by a two-way ANOVA followed by LSD test. Each of the two factors had two levels in every case: for whitefly species, MEAM1 and MED; and for temperature, a high temperature and a control temperature. The differences between treatments were considered significant when *P *< 0.05. All statistical analyses were conducted using SPSS 20.0 Statistics and Excel.

## Results

### Survival of MEAM1 and MED eggs following exposure to high temperatures

**Figure 1:**
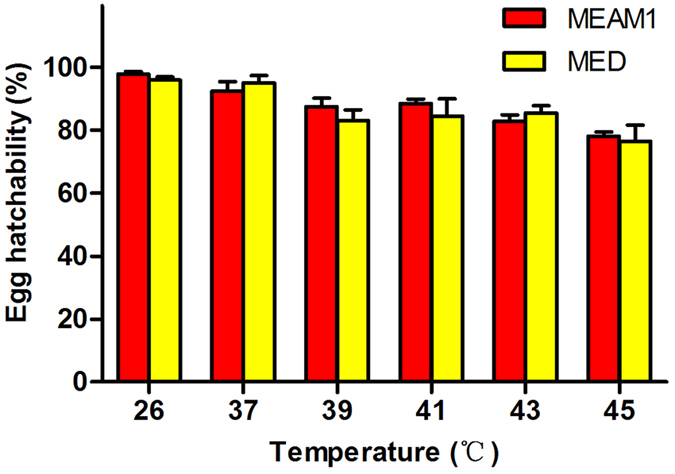
Performance of MEAM1 and MED eggs following exposure to high temperatures. Data are presented as mean ± SE of five replicates. In no case do the two mean percentages of egg hatch between the two whitefly species differ significantly as determined by independent-sample Student-t test (*P* > 0.05).

Compared to the egg hatch at the control temperature of 26 °C, the percentages of egg hatch following a 2 h exposure to high temperatures from 37 to 45 °C did not decline significantly; and in no case did the percentages of egg hatch differ between the two whitefly species at the same temperature regime (*P* > 0.05 in all cases; Fig. 1).

### Survival of MEAM1 and MED pupae following exposure to high temperatures

**Figure 2:**
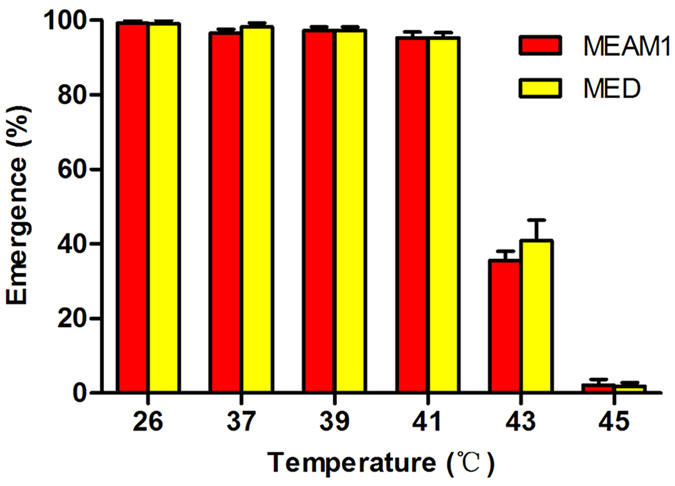
Performance of pupae following exposure to high temperatures. Data are presented as mean ± SE of five replicates. In no case do the two mean percentages of adult emergence between the two whitefly species differ significantly as determined by independent-sample Student-t test (*P* > 0.05).

Compared to the adult emergence at the control temperature of 26 °C, the percentages of pupal survival, i.e. adult emergence, did not decline following a 6 h exposure to high temperatures from 37 to 41 °C, in both MEAM1 and MED; however, a 6 h exposure to 43 °C resulted in subsequent decline of percentages of adult emergence from nearly 100% to about 40%, in both MEAM1 and MED, and a 6 h exposure to 45 °C resulted in the decline to close to zero, in both MEAM1 and MED (Fig. 2). Similar to the consequence of exposure of eggs to high temperatures, in no case did the percentages of adult emergence differ between the two whitefly species (*P* > 0.05 in all cases; Fig. 2).

### Survival of MEAM1 and MED adults following exposure to low temperatures

**Figure 3:**
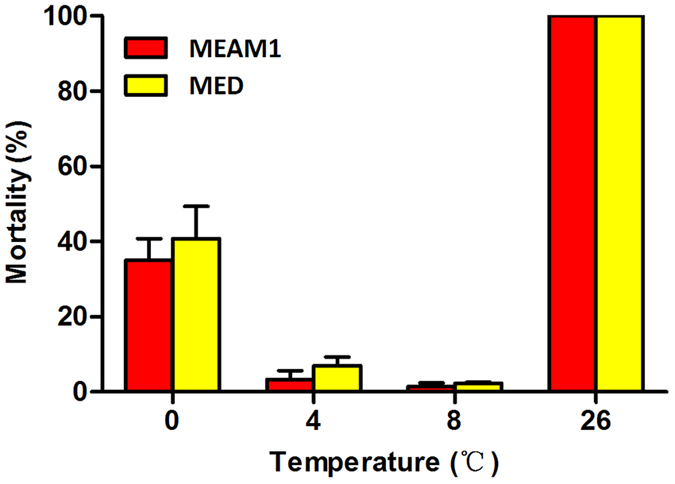
Performance of adults following exposure to low temperatures. Data are presented as mean ± SE of five replicates. In no case do the two mean percentages of adult mortality between the two whitefly species differ significantly as determined by independent-sample Student-t test (*P* > 0.05).

A 48 h exposure to the control temperature of 26 °C without food resulted in 100% mortality in both MEAM1 and MED (Fig. 3). These deaths following exposure to 26 °C were most likely caused by starvation rather than any effect of temperature as the same exposure to 26 °C with suitable host plants never resulted in >5% mortality. A 48 h exposure to 8 or 4 °C resulted in low levels of mortality, but a 48 h exposure to 0 °C resulted in about 40% mortality in both MEAM1 and MED (Fig. 3). However, in no case did the percentages of mortality differ between the two whitefly species (*P* > 0.05 in all cases; Fig. 3).

### Survival of MEAM1 and MED adults following exposure to high temperatures

**Figure 4:**
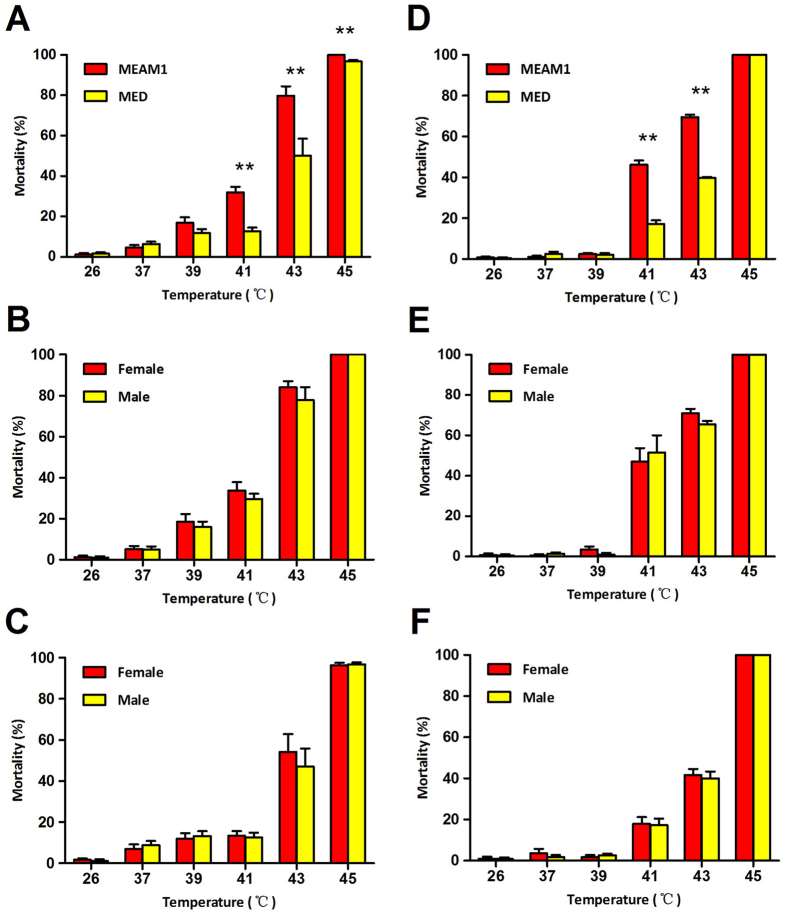
Performance of adults following exposure to high temperatures. Data are presented as mean ± SE of 10 replicates. (**A**) Percentages of mortality of adults of the two whitefly species from Zhejiang; **indicate significant difference between the two means at a given temperature (independent- samples Student-*t* test, *P* < 0.01). (**B**) Percentages of mortality of female and male adults of MEAM1from Zhejiang; in no case do the two mean percentages differ significantly (*P* > 0.05). (**C**) Percentages of mortality of female and male adults of MED from Zhejiang; in no case do the two mean percentages differ significantly (*P* > 0.05). (**D**) Percentages of mortality of adults of the two whitefly species from Guangdong; **indicate significant difference between the two means at a given temperature (*P* < 0.01). (**E**) Percentages of mortality of female and male adults of MEAM1 from Guangdong; in no case do the two mean percentages differ significantly (*P* > 0.05). (**F**) Percentages of mortality of female and male adults of MED from Guangdong; in no case do the two mean percentages differ significantly (*P* > 0.05).

In the populations collected from Zhejiang, the mortality of MEAM1 and MED adults increased with exposure to increasing temperatures, ranging from 2.5% at the control temperature of 26 °C to 96.0–100.0% at 45 °C (Fig. 4A). Following exposure to 37 and 39 °C, the percentages of mortality did not differ between MEAM1 and MED; however, the percentages of mortality of MEAM1 were significantly higher than those of MED following exposure to 41, 43 and 45 °C, respectively (*P* < 0.05 in all cases; Fig. 4A). At each of the exposure temperature, the percentages of mortality were similar between females and males, in both MEAM1 (*P* > 0.05 in all cases; Fig. 4B) and MED (*P* > 0.05 in all cases; Fig. 4C).

The data obtained with exposures of the Guangdong populations of MEAM1 and MED to the high temperatures were in general similar to those obtained with the Zhejiang populations of the two species. For example, following exposures to 41 and 43 °C, percentages of mortality were significantly higher in MEAM1 than in MED in both cases (*P* < 0.05 in all cases; Fig. 4A,D); and no significant differences in mortality were observed between the two sexes of MEAM1 from either Zhejiang or Guangdong (*P* > 0.05 in all cases; Fig. 4B,E), or between the two sexes of MED from either Zhejiang or Guangdong (*P* > 0.05 in all cases; Fig. 4C,F).

### Fecundity and performance of offspring of MEAM1 and MED following exposure of adults to low temperature

In both MEAM1 and MED, each of the four life history variables examined did not differ significantly between the exposure to 4 °C and control temperature of 26 °C (Table 1).

### Fecundity and performance of offspring of MEAM1 and MED following exposure to high temperature

In the populations from Zhejiang, a 2 h exposure to 41 °C resulted in significant reduction in the number of eggs laid, a marginal reduction of percentage of egg hatch, and a significant reduction in the female ratio in the offspring, in both MEAM1 and MED; however, neither whitefly species nor the interactions between the two factors had a significant effect on any of the life history variable examined (Table 2).

The data obtained with exposures of the Guangdong populations of MEAM1 and MED to the high temperature were essentially similar to those obtained with the Zhejiang populations of the two species, except that the reduction in percentage of egg hatch reached a significant level in both species (Table 2).

### Expression of heat shock protein related genes following exposure to high temperature

**Figure 5:**
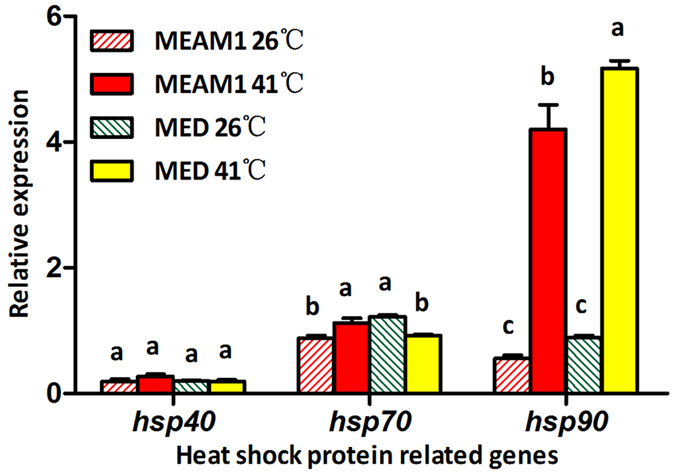
Levels of relative expression of heat shock protein related genes in MEAM1 and MED following exposure to high temperatures. Data are presented as mean ± SE of five replicates. Different letters above the four bars of each gene indicate significant differences between them as determined by a two-way ANOVA (LSD, P < 0.05).

Following a 2 h exposure to 41 °C, the level of expression of *hsp40* was unaffected by whitefly species, temperature, or the interaction between the two factors (Fig. 5, Table 3). The level of expression of *hsp70* was unaffected by neither whitefly species nor temperature, but was significantly affected by the interactions between the two factors (Fig. 5, Table 3). In contrast, the level of expression of *hsp90* was substantially affected by both whitefly species and temperature, but not by the interactions between the two factors; and the elevation of the level of expression of *hsp90* was more substantial in MED than that in MEAM1 (Fig. 5, Table 3).

## Discussion

In this study, we examined the survival and performance of eggs, pupae and adults of both MEAM1 and MED whiteflies following exposure to a range of high temperatures; we also examined the performance of the adults of the two species following exposure to low temperatures. Due to issues of technical feasibility, we were unable to examine the effects of exposure of eggs and pupae to low temperatures. In addition, part of the experiments was repeated using whitefly populations collected from two widely separated geographic locations. Our data demonstrated that the MED species was more tolerant to high temperatures than the MEAM1 species, especially in the adult stage (Fig. 4), and this difference in thermal responses between the two species may be related to the heat shock protein related genes, in particular *hsp90* and *hsp70* (Fig. 5 and Table 3). This result is consistent with that of previous reports^35^,^38^. Our data further show that thermal tolerance of egg and pupae to high temperatures were similar between the two whitefly species (Figs 1 and 2). Trials of exposure to low temperatures did not reveal differences between MEAM1 and MED. However, the lowest temperature we tested was 0 °C for 48 h and the mortality for both species was only 40% (Fig. 3). Thus the lack of differences between the two species was likely due to experimental set-up that did not include temperatures sufficiently low to show the differences between the two whiteflies.

While the data presented here indicate that populations of the MEAM1 or MED from Zhejiang and Guangdong showed similar levels of tolerance to high temperatures, our experiments included whitefly populations from only two geographic locations and no strict comparison of populations of the same species from the two locations was made. Thus, possible variations in temperature tolerance between geographic populations of the same whitefly species must not be neglected in future studies.

When the data obtained in this study were viewed together with those of previous reports^34^,^35^,^36^,^37^, for the populations of MEAM1 and MED examined so far, the lethal high temperature to adults was around 43–44 °C for MEAM1 and around 45–46 °C for MED, although slight differences between populations of the same species may occur. In the regions of Zhejiang and Guangdong where the test populations were originally collected, maximal temperatures in the summer in the field may often go up to 40–45 °C, and in Zhejiang the minimum temperatures in the winter often approaches 0 to −2 °C. These high temperatures of 40–45 °C may also occur in glasshouses in the summer in all regions throughout China. Thus the higher tolerance of MED to high temperatures than MEAM1 may offer advantages to the former species in distribution and abundance when critical high temperatures are experienced in the field or greenhouses by the two species of whiteflies.

The observation that MED is more tolerant to high temperatures than MEAM1 has important implications in understanding the distribution and seasonal abundance of the two whitefly species in the field. However, application of this finding to predict the performance of the two species in the field requires caution, as thermal tolerance of whiteflies may be affected by many other factors, such as host plants^30^,^32^,^33^, endosymbiont community of whiteflies^43^ and plant virus-carrying status of whiteflies^44^. Another important factor that requires particular attention in using laboratory findings to understand or predict field performance of the whiteflies is that evolutionary changes of thermal response or tolerance may occur fairly rapidly in invasive species such as MEAM1 and MED whiteflies^44^,^45^,^46^.

## Additional Information

**How to cite this article**: Xiao, N. *et al.* Differential tolerance capacity to unfavourable low and high temperatures between two invasive whiteflies. *Sci. Rep.* **6**, 24306; doi: 10.1038/srep24306 (2016).

## Figures and Tables

**Table 1:** Fecundity and performance of offspring of MED and MEAM1 whiteflies following exposure of the adults to a low temperature.

Life history variables					Two-way ANOVA statistics					
	MEAM1		MED		Whitefly species		Temperature		Interactions	
	26 °C	4 °C	26 °C	4 °C	*F*_1,10_	*P*	*F*_1,10_	*P*	*F*_1,10_	*P*
No. of eggs producedper female	6.5 ± 0.7	5.2 ± 0.2	5.8 ± 0.4	6.1 ± 1.4	0.017	0.899	0.380	0.551	2.501	0.378
Percentage of egghatch	99.0 ± 0.5	98.1 ± 0.7	97.8 ± 1.4	97.9 ± 1.0	0.243	0.632	0.193	0.670	0.120	0.763
Development timein days from egg toadult of offspring	21.5 ± 0.7	21.5 ± 0.2	22.4 ± 0.5	22.4 ± 0.4	4.247	0.066	0.000	0.999	0.007	0.936
Percentage of femalesin offspring	46.8 ± 4.6	56.2 ± 5.3	41.7 ± 2.5	47.1 ± 6.3	5.089	0.048	0.558	0.472	0.011	0.917

Data are presented as mean ± standard error for five replicates. In no case do the four means on the same line of a given life history variable of the two whitefly species under two temperatures differ significantly as determined by a two-way ANOVA (*P* > 0.05).

**Table 2:** Fecundity and performance of offspring of MED and MEAM1 whiteflies following exposure of the adults to a high temperature.

Life history variables					Two-way ANOVA statistics					
	MEAM1		MED		Whitefly species		Temperature		Interactions	
	26 °C	41 °C	26 °C	41 °C	*F*_1,12_	*P*	*F*_1,12_	*P*	*F*_1,12_	*P*
**Zhejiang populations**										
No. of eggs produced per female	7.1 ± 1.0 a	2.9 ± 1.2 b	6.2 ± 0.9 a	3.3 ± 0.1 b	0.065	0.802	**15.818**	**0.002**	0.485	0.500
Percentage of egg hatch	98.8 ± 0.9 a	94.4 ± 2.6 a	99.3 ± 0.4 a	95.2 ± 2.0 a	0.072	0.793	**4.691**	**0.051**	0.000	0.999
Development time in days from egg to adult of offspring	21.1 ± 0.2 a	21.9 ± 0.4 a	22.2 ± 0.7 a	22.6 ± 0.6 a	2.305	0.155	0.829	0.380	0.532	0.480
Percentage of females in offspring	58.4 ± 3.8 a	42.0 ± 5.1 b	55.3 ± 5.3 ab	46.3 ± 3.9 ab	0.053	0.819	**6.625**	**0.015**	0.698	0.410
**Guangdong populations**										
No. of eggs produced per female	5.7 ± 0.7 a	2.2 ± 0.6 b	7.1 ± 1.2 a	3.1 ± 0.7 b	1.785	0.206	**22.408**	**0.001**	0.078	0.785
Percentage of egg hatch	98.4 ± 0.9 a	90.7 ± 1.5 b	97.4 ± 1.1 a	88.8 ± 3.4 b	0.569	0.465	**19.243**	**0.001**	0.036	0.852
Development time in days from egg to adult of offspring	20.9 ± 0.2 a	21.1 ± 0.7 a	21.3 ± 0.4 a	22.0 ± 0.3 a	2.319	0.154	1.385	0.262	0.257	0.621
Percentage of females in offspring	61.5 ± 7.5 a	37.8 ± 4.5 b	56.1 ± 3.3 a	41.8 ± 12.2 b	0.034	0.857	**5.814**	**0.033**	0.189	0.672

Data are presented as mean ± standard error for 10 replicates. The four means on the same line of a given life history variable of the two whitefly species from Zhejiang or Guangdong differ significantly when followed by different lower case letters (*P* < 0.05).

**Table 3:** Two-way ANOVA statistics on the expression of heat shock protein related genes of MEAM1 and MED following exposure to high temperature.

Genes	Whitefly species		Temperature		Interactions	
	*F*_1,12_	*P*	*F*_1,12_	*P*	*F*_1,12_	*P*
*hsp40*	1.644	0.224	1.695	0.217	2.531	0.138
*Hsp70*	3.261	0.150	0.358	0.561	**33.111**	**<0.001**
*Hsp90*	**9.876**	**0.008**	**363.354**	**<0.001**	2.347	0.151
